# Whole Genome Resequencing Helps Study Important Traits in Chickens

**DOI:** 10.3390/genes14061198

**Published:** 2023-05-30

**Authors:** Xinwei Xiong, Jianxiang Liu, Yousheng Rao

**Affiliations:** Key Laboratory for Genetic Improvement of Indigenous Chicken Breeds of Jiangxi Province, Nanchang Normal University, Nanchang 330032, Chinarys8323571@aliyun.com (Y.R.)

**Keywords:** whole genome resequencing, chicken, qualitative traits, quantitative traits, adaptability and disease resistance

## Abstract

The emergence of high-throughput sequencing technology promotes life science development, provides technical support to analyze many life mechanisms, and presents new solutions to previously unsolved problems in genomic research. Resequencing technology has been widely used for genome selection and research on chicken population structure, genetic diversity, evolutionary mechanisms, and important economic traits caused by genome sequence differences since the release of chicken genome sequence information. This article elaborates on the factors influencing whole genome resequencing and the differences between these factors and whole genome sequencing. It reviews the important research progress in chicken qualitative traits (e.g., frizzle feather and comb), quantitative traits (e.g., meat quality and growth traits), adaptability, and disease resistance, and provides a theoretical basis to study whole genome resequencing in chickens.

## 1. Introduction

Chickens are important in the egg and meat industries and are widely distributed worldwide. The most valuable traits of chicken include growth, carcass, and meat quality. Each trait is complex and affected by multiple factors, including environmental, such as feeding, management, and slaughter conditions, and genetic backgrounds, such as breed, gene, and gene interactions. Improvements in phenotypic selection have increased production parameters, such as the body weight and growth rate of chickens. However, non-uniformity in performance within and between chicken breeds remains an issue [1]. Moreover, the phenotypes of the carcass and meat quality traits can only be recorded after slaughter, precluding the selection of breeding individuals based on these traits [2]. Therefore, understanding the molecular basis of inter-individual variation in traits provides valuable information on their genetic architecture and helps improve these traits in chicken breeding.

The rise in the generation of high-throughput and low-cost sequencing platforms has led to an increasing number of livestock samples analyzed by high-throughput sequencing. In recent decades, an increasing number of quantitative trait loci (QTL) were detected for economically important traits in livestock through genetic linkage analysis [3]. In particular, the use of the generation sequencing platform to resequence the representative individuals of different groups within a species containing a known genome can quickly and efficiently obtain genome information and move current genomics research into the era of genome resequencing. In 2004, de novo sequencing of the whole genome of red junglefowl Gallus gallus marked the beginning of chicken genomic research [4]. The availability of high-throughput whole genome resequencing has allowed for the detection of significant associations of nucleotide polymorphisms with complex traits and causal genes and mutations for monogenic traits through genome-wide association studies (GWAS) and/or selection signature analysis [5,6,7] to provide the foundation for molecular-assisted chicken breeding.

The chicken is an ideal model organism for molecular genetics and human medical research [8]. Some important economic traits, such as egg quality, meat quality, and growth traits, have been widely studied following increasing human demand and green aquaculture. This review describes the factors influencing whole genome resequencing and their differences compared with whole genome sequencing. We also summarize the important research progress in chicken qualitative traits, quantitative traits, adaptability, and disease resistance.

## 2. Overview of Whole Genome Resequencing

### 2.1. Definition of Whole Genome Resequencing

Whole genome resequencing refers to the sequencing of different individuals and populations of a species on the premise that its whole-genome sequence is known [9]. Whole genome resequencing analysis comprehensively analyzes the measured genome data and compares the obtained sequence with the reference sequence to determine differences in the whole genome. To date, many nucleotide polymorphisms, short insertions/deletions (InDels), structural variations (SVs), and copy number variations (CNVs) have been obtained through whole genome resequencing. Bioinformatic methods are then used to process the data, analyze the differences in the gene structure of diverse individuals or groups, and mark their variation sites. This laid a data foundation for advanced analytical fields, including GWAS, population evolution analysis, and breeding selection. Moreover, Bourgeois and Warren summarized current population genomic methods for the analysis of whole genome resequencing data in eukaryotes and discussed some of their limitations [10].

### 2.2. Factors Affecting Whole Genome Resequencing

In this part, the major factors influencing whole genome resequencing, such as sequencing depth, coverage, and platform, were included.

#### 2.2.1. Sequencing Depth and Coverage

Sequencing depth refers to the ratio of the total number of bases obtained by sequencing to the genome size [11]. A greater sequencing depth lowers the probability of false positive sequencing results. Sequencing coverage refers to the proportion of base coverage obtained by genome sequencing and reflects the randomness of sequencing; it is positively correlated with sequencing depth [11]. Different sequencing depths and coverage yields different results. A sequencing depth below 4× only covers 95% of the whole genome, and the number of false positive variants is larger compared with a sequencing depth of 10× that can reach 99% of the whole genome and result in a plateau [12]. Therefore, 10× is the ideal sequencing depth to achieve platform coverage and accurately identify mutations [12].

#### 2.2.2. Sequencing Platform

Genome sequencing has progressed from the first generation of DNA end-termination sequencing (Sanger sequencing) to the second generation of high-throughput sequencing (HTS) and the third generation of single-molecule sequencing, such as Nanopore sequencing [13,14,15,16,17]. Second-generation sequencing technology is rapid, low-cost, and generates a large amount of data. Third-generation sequencing technology has ultra-high read length, high throughput, and a short sequencing time. Second- and third-generation sequencing technologies are mainly used for whole genome resequencing. Different platforms may have systematic differences in the quality of raw data, comparison quality, and detection of single nucleotide polymorphism (SNP) variations. The sequencing data of MGISEQ-2000 and HiSeq-2500 are similar in terms of the original data quality and variation detection; however, the comparison quality of MGISEQ-2000 is better than that of HiSeq-2500 [18]. Whole genome resequencing analysis has shown that MGISEQ-2000 is superior to HiSeq-2000 and NovaSeq-6000 in terms of the ratio of repeat reads and quality of comparison and has higher accuracy of SNP variation detection than NovaSeq-6000 [19]. Researchers can select resequencing platforms that meet the data quality experimental requirements at a lower price to obtain high-quality data.

### 2.3. Differences between Whole Genome Resequencing and Whole Genome Sequencing

The differences between whole genome sequencing and whole genome resequencing include sequencing objects, research purposes, requirements for sequencing depth, and differences in the content of bioinformatics analysis. First, the objective of whole genome sequencing is to identify a species with an unknown genome sequence to obtain its whole-genome sequence. In contrast, whole genome resequencing is performed to determine the genomes of different individuals or groups on the premise that the whole genome sequence of the species is known. The genome sequence can be acquired through sequence splicing even if the sequencing depth is shallow; therefore, it consumes less than whole genome sequencing. Second, the purpose of whole genome sequencing is to obtain the whole genome sequence of a species to understand its genome size and complexity and to conduct relevant data processing, such as labeling its trait genes and mapping genes. The objective of whole genome resequencing is to compare the sequencing results of different individuals of the same species with those of other samples or chips to find a large number of SNPs, InDels, SVs, and CNVs, and to complete the annotation of relevant genes. Whole genome sequencing needs to reach 50–100× of sequencing depth using second-generation sequencing technology; however, the resequencing depth only needs to reach 10–30× to ensure that there is an acceptable genome coverage rate and sequencing error rate. Finally, the main content of whole genome sequencing is genome assembly and annotation using bioinformatics analysis. In contrast, bioinformatic analysis of whole genome resequencing mainly focuses on individual SNPs, InDels, and other heritable variants and involves genetic evolution analysis, trait gene prediction, and animal breeding.

## 3. Application of Whole Genome Resequencing in Chickens

After long-term natural selection and artificial domestication, chickens currently show rich genetic diversity in morphology, structure, behavior, physiology, and diseases [20]. These various traits can be summarized into two categories according to their manifestation: qualitative and quantitative traits. However, there is a special type of trait called a threshold trait, which combines some of the characteristics of qualitative and quantitative traits. Its manifestation shows discontinuous variation but does not follow Mendelian genetic laws. It is generally believed that this type of trait has a potential continuous variable distribution and that its genetic basis is similar to that of quantitative traits. This special trait type of trait is controlled by multiple genes, such as disease resistance and adaptability. In this section, we mainly review the application of whole genome resequencing in these three types of traits (Table 1).

### 3.1. Application in the Qualitative Traits of Chickens

Qualitative traits refer to traits that clearly distinguish different phenotypes but are not quantitatively observed. It is generally believed that there is no continuous quantitative relationship between different phenotypes, including combs, feather colors, and frizzle feathers.

#### 3.1.1. Frizzle Feather

Whole genome resequencing in four populations of three chicken breeds identified a missense mutation (g.5281494A>G) and a 15-bp deletion (g.5285437-5285451delGATGCCGGCAGGACG) in KRT75L4 as candidate gene and mutations in Xiushui yellow chickens through comparative genomics and GWAS analysis to determine the molecular mechanism of frizzle feathers [21]. Moreover, two candidate mutations were confirmed in a large Xiushui yellow chicken population, and a 15-bp deletion in KRT75L4 was identified as the putative causative mutation of the frizzle feather [21]. Three independent selection signal analyses of Qilin chickens showed that the GGA33:1.17M-1.39M region was a significant selection region shared by the three detection methods based on whole genome resequencing data [46]. A 15-bp deletion mutation in the KRT75L4 gene may also control the formation of the chicken frizzle feather phenotype [46]. However, this result was inconsistent with a report showing that a 69-bp deletion in KRT75 was responsible for the frizzle phenotype in chickens [22]. Therefore, there may be breed-specific differences in the control of genes related to frizzle feathers among different chicken breeds.

#### 3.1.2. Comb

The comb is an important feature of chickens that is primarily composed of collagen and hyaluronan and is produced by chondrocytes. A previous whole-genome resequencing and linkage analysis has revealed an increase in the copy number in a non-coding sequence of the SOX5 gene associated with the phenotype of pea-comb and identified the molecular mechanism of pea-comb formation [23]. In 2019, a whole genome resequencing study on the characteristics of the Araucana chicken breed in North America showed that eight significant selection regions and 203 SNPs were related to the pea-comb, blue eggshell, and rumplessness traits [47]. Using 60 K chicken iSelect chip data and whole-genome resequencing data, a 20 Kb duplication was identified at 200 Kb upstream of the EOMES gene, which is only present in chickens that have a duplex-comb phenotype [24]. In addition, sequence reversal of 7.4 Mb on chromosome 7 can cause transient ectopic expression of the MNR2 homeodomain protein gene that is related to the rose-comb [25].

#### 3.1.3. Feather Color and Other Traits

Feather colors and other qualitative traits are more intuitive and obvious phenotypes that perform an important role in breed identification in chickens. Resequencing of the whole genome of 86 domestic chickens representing 10 phenotypically diverse breeds showed that the PDSS2 gene was the cause-and-effect gene of silk feathers in Jinyang silky chickens and that the EDN3, MC1R, TUBB1, and TUBB3 genes might be related to the black skin, black bone, and black feather traits [26]. However, resequencing the whole genome of Baicheng You chickens with black, lavender, and yellow plumage colors showed that EGR1 is the most plausible candidate gene for black plumage; RAB17, MLPH, and SOX5 are likely genes for lavender plumage; and GRM5 is a candidate for yellow plumage [27]. Resequencing the whole genomes of 100 yellow-feathered chickens from 10 major traditional breeds and 10 Huaibei partridge chickens from China resulted in a consistent BCDO2 haplotype differentiation pattern among these different, yellow-feathered chicken breeds, which was consistent with the haplotype differentiation pattern of other yellow-feathered chicken candidate genes. This result confirmed that the BCDO2 gene is the main candidate gene for yellow pigment deposition [28]. Moreover, whole genome resequencing, genome-wide association analysis, and identity-by-descent (IBD) analyses revealed that a complex structural variation on chicken chromosome 27 (GGA27), composed of three CNV rearrangements, causes chicken muffs and beard phenotype [29]. The structural variation affected the ectopic expression of HOXB8, resulting in chicken muffs and a beard phenotype [29]. In addition, a whole genome resequencing analysis revealed genetic indels of a feathered-leg trait in domestic chickens [30]. Gene function annotation and analysis of quantitative trait loci identified 24 potential candidate genes affecting the feathered-leg trait, with FGF3 and FGF8 considered as the two main genes [30]. Moreover, whole-genome sequencing, expression analyses, and comparative genomics of 169 chicken individuals identified that the 17-kb deletion mutation upstream of the PITX1 gene and a non-coding mutation upstream of the TBX5 gene (GGA15: 12573054T>C) led to the formation of the chicken feathered-leg trait [31].

### 3.2. Application in the Quantitative Traits of Chickens

Quantitative phenotype traits, including growth, meat quality, and reproductive traits, are measurable and continuously change.

#### 3.2.1. Meat Quality

Traits, including meat color, pH, and drip loss, are important economic traits used to evaluate meat quality. They are influenced by factors, such as breed, sex, feeding methods, nutrition, and the metabolic level of the body. A genome-wide association study of muscle glycogen traits based on whole genome resequencing data of 474 Jingxing yellow chickens showed that nine SNPs and three indels were significantly related to muscle glycogen metabolism [48]. Among these, CPNE4 and NKD1 were key candidate genes affecting the process of glucose metabolism. In addition, 10× whole-genome resequencing analysis of 200 Danzhou chickens has shown that ten SNPs were associated with meat color b *, which were distributed on chromosomes 1, 2, and 4; four SNPs were associated with meat color a *, which were distributed on chromosomes 1, 2, and 5; five SNPs were associated with meat color L *, which were distributed on chromosomes 1, 2, and Z [32]. Moreover, the ACSS3 and ACAA2 genes were revealed to be candidate genes for Danzhou chicken color traits through Gene Ontology (GO) and Kyoto Encyclopedia of Genes and Genomes (KEGG) analyses [32]. Whole genome resequencing to screen for specific missense SNPs in high and low meat color chicken strains identified 15 candidate genes affecting chicken color traits after gene annotation and functional enrichment analysis [49]. Whole genome resequencing of 28 chickens from Brazilian broiler and layer strains obtained a total of 13.93 million SNPs and 1.36 million indels. SNPs and indels data were screened and identified using the sliding window strategy F_ST_ selection signal analysis to identify 8 candidate genes related to muscle development and 12 candidate genes related to fat deposition [50]. Furthermore, whole genome resequencing of three purebred broilers (n = 748) and six local breeds/lines (n = 114), transcriptome sequencing data of six tissues from two chicken breeds (n = 129), and whole genome resequencing data from an online database of twelve chicken breeds (n = 199) showed that the MYH1 gene family had the highest selection signatures and muscle-specific expression in purebred broilers, whereas the SOX6 gene influenced breast muscle yield and was also related to myopathy [33].

#### 3.2.2. Reproductive Traits

Reproductive traits, including the number of eggs laid, egg weight, and egg quality, are important economic traits for poultry. Exploring the mechanisms of reproductive trait-related genes in poultry is crucial to improve the reproductive performance of poultry. The selection of four pendulous-comb and four upright-comb chickens at 217 days of age for RNA-seq and whole genome resequencing identified CAMK1D, CLSTN2, MAST2, PIK3C2G, TBC1D1, STK3, ADGRB3, and PPARGC1A as potential candidate genes for egg production [34]. The whole genome resequencing analysis identified 37 SNPs on GGA20 that were associated with egg production in chickens, and gene annotation revealed 37 SNPs were identified in the BMP7 gene [35]. A previous study has revealed a significant correlation between the KIF18A and LIN28 genes and egg production in local Chinese chicken breeds through whole genome resequencing [26]. Furthermore, mixed pool genomic resequencing analysis based on the phenotypic data of eggshell strength and thickness in two groups of chickens identified CACNA1H and its non-synonymous mutation (g.5306181T>C) as candidate gene and mutation affecting eggshell thickness and strength [36]. The authors also successfully identified the CACNA1H gene as a key gene for eggshell calcification through population validation experiments [36].

#### 3.2.3. Growth Traits

Growth traits are important economic traits in chickens and were mostly selected through artificial breeding. Bioinformatic technology and molecular marker-assisted breeding can be used to shorten the breeding process and improve breeding efficiency. Whole genome resequencing analysis and population verification of Xichuan chickens identified two indels (52 bp and 224 bp) in the promoter region of the chicken QPCTL gene that were significantly related to chicken weight and ketone body traits [37]. The QPCTL gene can be used as a molecular genetic marker for chicken breeding. Resequencing technology was used to correlate the 86 bp indel downstream region of the MLNR gene with the growth traits of chickens [38]. The authors determined a significant correlation between the homozygous DD genotype and fast-growing chickens after genotyping over 2000 individuals from 9 different breeds. Another study using whole genome sequencing of unique local chicken in Yunnan-Dulong chicken identified 469 important candidate genes [51]. The authors found that the FAM19A5 gene was related to chicken body size; the result was consisted with a study of beef cattle [39]. Furthermore, comparative genome analysis and ROH analysis of normal-sized chicken embryos and embryos with autosomal dwarfism showed that the c.433G>A nonsense mutation site of the TMEM263 gene caused chicken autosomal dwarfism using whole genome resequencing [40]. TMEM263 interacts with growth hormone (GH1) and bone morphogenetic protein (BMP2) genes to affect chicken bone growth.

### 3.3. Application in Adaptability and Disease Resistance of Chickens

Chickens have been economically important to humans since ancient times. Numerous heritable variations are produced after long-term natural and artificial selection. Whole genome resequencing technology can explore adaptive changes in chickens and reveal their population genetic mechanisms. Researchers can predict the direction of chicken population evolution, and artificial intervention can result in the evolution of chickens that are beneficial to humans by preventing genetic diseases and obtaining more economic benefits. General phenotypic genes, economic trait genes, disease-causing genes, and other gene loci determined by whole genome resequencing and follow-up studies can provide key information on animal molecular breeding research. This is one of the keys to breeding science.

Whole genome resequencing data of 787 red junglefowl subspecies from different geographical ranges and assumed wild-related populations worldwide were resequenced and integrated with the published genome information of 76 chickens [52]. Systematic evolution, principal component analysis, and population structure analyses conducted on these 863 genomes determined that the domestic chicken originated from a subspecies of the Red junglefowl chicken, Gallus gallus spadiceus, mainly distributed in southwestern China, northern Thailand, and Myanmar, then transferred to Southeast and South Asia for reproduction due to changes in the natural environment [52]. In 2015, de novo sequencing of a female Tibetan chicken genome and whole genome resequencing of 5 red jungle chickens and 27 other domestic chickens of different breeds (10 Tibetan chickens, 8 Yunnan native chickens, 8 Xishuangbanna fighting chickens, and 1 Roman hen) was performed [53]. The results indicated that the utilization of genetic resources in local Chinese chicken breeds had reached a new high level. Population and comparative genomics revealed the genetic mechanism of the adaptive evolution of Tibetan chickens at high altitudes and hypoxia; Tibetan chickens were divided into two groups without gene exchange, and it was inferred that the two Tibetan chickens might have originated from independent lineages. The strongly selected genes in all Tibetan chicken populations were mainly enriched in the calcium ion signaling pathway, as revealed by detecting the effective population size and selection signal of all Tibetan chicken populations. This result indicated that the calcium ion pathway might perform an important role in the high-altitude hypoxia adaptation of Tibetan chickens. In 2023, Shi et al. performed a whole genome resequencing analysis of 119 domesticated chickens in China, including Wenchang chicken, green-shell chicken, Tibetan chicken, and Lindian chicken, to determine the genetic signatures of their adaptation to tropical and frigid environments [41]. The results showed that the Wenchang chicken branched off earlier than the Lindian chicken with an evident genetic admixture, suggesting their closer genetic relationship and that the SLC33A1 and TSHR genes exhibited stronger signatures for positive selection in the genome of the more tropical Wenchang chicken. The genes, which are related to the unique characteristics of the inbreeding of yellow feather chickens, have been identified in a previous study aiming to detect the genomic degree of purity (ROH) using whole genome resequencing of 10 yellow feather chickens from 10 different yellow feather chicken breeds [54]. A total of 25,547 ROH were detected with an average length of 335 kb, most of which were below 1 Mb. This result indicated that shorter ROH performed a dominant role in the breeding history of this chicken species. The number, length, frequency, and distribution of ROH differed in chicken populations with high genetic diversity. In addition, the IFNA, IFNB, IL11RA, IL22RA1, IFNLR1, and TRIF genes were related to the disease resistance mechanism of yellow feather chickens. In a previous study, whole genome resequencing showed that some Japanese quails genes were mixed into red jungle fowl and 15 domestic chicken populations after a long period of cross-breeding, and genes were mixed between Tibetan chicken, wild red jungle fowl, and other chicken breeds [42]. The genetic diversity of domestic chickens has decreased in recent years owing to long-term inbreeding under artificial breeding conditions. Nowadays, human activities have facilitated mating between Tibetan and domestic chickens (from k = 2 to k = 7), which is conducive to artificial breeding and protecting genetic diversity. Many genes with strong selective clearing signals in the genome are involved in growth, metabolism, immunity, behavior, and reproductive signaling pathways. Whole genome resequencing technology has been used to construct full sib F1 to reveal the allelic transmission rate-distortion (TRD) sites in chickens [55]. Two parents of the chickens were used for 30× deep resequencing, and 38 of their offspring were used for 5× deep resequencing. The TRD locus in chicken chromosome 16 performed an important role in chicken immunity and disease resistance. The MYH1F gene significantly affects TRD allele-specific expression, which performs a key role in the rapid development of chicken muscle. Functional enrichment analysis showed that many TRD-related genes (such as TGFBR2, TGFBR3, NOTCH1, and NCOA1) were related to embryo death and germline selection. This study provided important data for further research on chicken disease resistance. Whole genome resequencing of Arkansas regression line (AR) chickens at 14× depth confirmed tumor regression characteristics compared with Arkansas progressive line (AP) chickens at 11× depth [43]. 63 SNP candidate loci were associated with tumor regression characteristics in AR chickens from 7.3 million and 7.1 million SNP loci in AP and AR chickens, respectively. Network analysis revealed found that 30 genes were related to developmental disorders, genetic diseases, and metabolic diseases, including ABCA12, ACP6, ARSB, and ARSD; ANO10, BANK1, BCR, and 22 other genes were related to cell death and survival, development, and function of the blood system, and humoral immune response; and 22 genes were associated with developmental disorders, gastrointestinal diseases, and genetic diseases, including ARRB1, BMX, and Cgm4/Psg16. Moreover, ubiquitination may regulate the stability of oncogenes or tumor suppressor proteins [44]. Amino acid changes in the proteins FAM208B (lysine residue changed to glutamic acid), LAMB4, and IFT140 (lysine residues changed to arginine residue) may indicate that the process of cellular protein degradation by changing their ubiquitination characteristics may perform an important role in tumor regression of AR chickens. PIK3C2G is an important component of the PI3K pathway. In AR chicken, the PI3K and NF-ĸB signaling pathways are downregulated due to an SNP in the PI3K pathway, resulting in apoptosis by the inhibition of tumors by p53. Furthermore, whole genome resequencing of susceptible and tolerant populations of broiler ascites disease showed that the CPQ gene is related to the resistance of ascites disease and is a key gene leading to broiler ascites disease [45].

## 4. Conclusions

Whole genome resequencing technology has led to significant achievements in chicken research in recent years and has rapidly developed in other biological research fields. Chickens occupy an irreplaceable position in the modern breeding industry, representing a high-quality meat food used as a human protein supplement and an excellent model organism in life science research. Resequencing the whole chicken genome can promote the protection and breeding of chicken breeds, which lays the foundation for scientific and technological progress. The continuous introduction of various whole genome sequencing platforms and improvements in sequencing technology can solve many problems encountered in sequencing. In particular, joint multi-omics analysis has become a more comprehensive and systematic solution. It is believed that improving sequencing technologies will achieve better results in chicken genomics, proteomics, epigenetics, and other fields in the future.

## Figures and Tables

**Table 1:** The genes identified to association with chicken traits through whole genome resequencing.

Taxa	Traits	Genes	References
Qualitative traits	Frizzle feather	KRT75L4, KRT75	Chen et al. (2022) [21], Ng et al. (2018) [22]
	Comb	SOX5, EOMES, MNR2	Wright et al. (2009) [23], Dorshorst et al. (2015) [24], Imsland et al. (2012) [25]
	Feather color	PDSS2, EDN3, MCIR, TUBB1, TUBB3, EGR1, RAB17, MLPH, SOX5, GRM5, BCDO2	Li et al., (2019) [26], Wang et al., (2022) [27], Huang et al., (2020) [28]
	Muffs and beard	HOXB8	Guo et al., (2016) [29]
	Feathered-leg	FGF3, FGF8, PITX1, TBX5	Yang et al., (2019) [30], Bortoluzzi et al., (2020) [31]
Quantitative traits	Meat quality	CPNE4, NKD1, ACSS3, ACAA2, MYH1, SOX6	Jiang et al., (2022) [32], Tan et al., (2023) [33]
	Reproductive traits	KIF18A, LIN28, CAMK1D, CLSTN2, MAST2, PIK3C2G, TBC1D1, STK3, ADGRB3, PPARGC1A, BMPT, CACNA1H	Li et al., (2019) [26], Cai et al., (2023) [34], Dong et al., (2019) [35], Zhang et al., (2015) [36]
	Growth traits	QPCTL, MLNR, FAM19A5, TMEM263, GH1, BMP2	Ren et al., (2019) [37], Liu et al., (2019) [38], An et al., (2019) [39], Wu et al., (2018) [40]
Threshold traits	Adaptability and disease resistance	SLC33A1, TSHR, IFNA, IFNB, IL11RA, IL22RA1, IFNLR1, TRIF, MYH1F, TGFBR2, TGFBR3, NOTCH1, NCOA1, ABCA12, ACP6, ARSB, ARSD, AN010, BANK1, BCR, ARRB1, BMX, FAM208B, LAMB4, IFT140, PIK3C2G, CPQ	Shi et al., (2023) [41], Chen et al., (2018) [42], Khatri et al., (2018) [43], Popovic et al., (2014) [44], Dey et al., (2018) [45]

## Data Availability

Not applicable.

## References

[1] Bindari Y.R., Gerber P.F. (2022). Centennial Review: Factors affecting the chicken gastrointestinal microbial composition and their association with gut health and productive performance. Poult. Sci..

[2] Xiong X., Zhou M., Zhu X., Tan Y., Wang Z., Gong J., Xu J., Wen Y., Liu J., Tu X. (2022). RNA Sequencing of the Pituitary Gland and Association Analyses Reveal PRKG2 as a Candidate Gene for Growth and Carcass Traits in Chinese Ningdu Yellow Chickens. Front. Vet. Sci..

[3] Hu Z.L., Park C.A., Wu X.L., Reecy J.M. (2013). Animal QTLdb: An improved database tool for livestock animal QTL/association data dissemination in the post-genome era. Nucleic Acids Res..

[4] International Chicken Genome Sequencing Consortium (2004). Sequence and comparative analysis of the chicken genome provide unique perspectives on vertebrate evolution. Nature.

[5] Rubin C.J., Zody M.C., Eriksson J., Meadows J.R., Sherwood E., Webster M.T., Jiang L., Ingman M., Sharpe T., Ka S. (2010). Whole-genome resequencing reveals loci under selection during chicken domestication. Nature.

[6] Hirschhorn J.N., Daly M.J. (2005). Genome-wide association studies for common diseases and complex traits. Nat. Rev. Genet..

[7] Jensen J.D., Wong A., Aquadro C.F. (2007). Approaches for identifying targets of positive selection. Trends Genet..

[8] Burt D.W. (2007). Emergence of the chicken as a model organism: Implications for agriculture and biology. Poult. Sci..

[9] Bentley D.R. (2006). Whole-genome re-sequencing. Curr. Opin. Genet. Dev..

[10] Bourgeois Y.X.C., Warren B.H. (2021). An overview of current population genomics methods for the analysis of whole-genome resequencing data in eukaryotes. Mol. Ecol..

[11] Sims D., Sudbery I., Ilott N.E., Heger A., Ponting C.P. (2014). Sequencing depth and coverage: Key considerations in genomic analyses. Nat. Rev. Genet..

[12] Jiang Y., Jiang Y., Wang S., Zhang Q., Ding X. (2019). Optimal sequencing depth design for whole genome re-sequencing in pigs. BMC Bioinform..

[13] Sanger F. (1988). Sequences, sequences, and sequences. Annu. Rev. Biochem..

[14] Shendure J., Ji H. (2008). Next-generation DNA sequencing. Nat. Biotechnol..

[15] Chen W., Kalscheuer V., Tzschach A., Menzel C., Ullmann R., Schulz M.H., Erdogan F., Li N., Kijas Z., Arkesteijn G. (2008). Mapping translocation breakpoints by next-generation sequencing. Genome Res..

[16] Yang J., Ferranti D.C., Stern L.A., Sanford C.A., Huang J., Ren Z., Qin L.C., Hall A.R. (2011). Rapid and precise scanning helium ion microscope milling of solid-state nanopores for biomolecule detection. Nanotechnology.

[17] Marshall M.M., Yang J., Hall A.R. (2012). Direct and transmission milling of suspended silicon nitride membranes with a focused helium ion beam. Scanning.

[18] Korostin D., Kulemin N., Naumov V., Belova V., Kwon D., Gorbachev A. (2020). Comparative analysis of novel MGISEQ-2000 sequencing platform vs Illumina HiSeq 2500 for whole-genome sequencing. PLoS ONE.

[19] Li W., Liu G., Zhou R., Tang Z., Liu J., Sun F. (2021). Comparative analysis of the whole genome resequencing data of MGISEQ-2000, HiSeq 2000 and NovaSeq 6000 platforms. J. Chin. Anim. Husb..

[20] Narita Y., Kuratani S. (2005). Evolution of the vertebral formulae in mammals: A perspective on developmental constraints. J. Exp. Zool. B Mol. Dev. Evol..

[21] Chen B., Xi S., El-Senousey H.K., Zhou M., Cheng D., Chen K., Wan L., Xiong T., Liao M., Liu S. (2022). Deletion in KRT75L4 linked to frizzle feather in Xiushui Yellow Chickens. Anim. Genet..

[22] Ng C.S., Wu P., Foley J., Foley A., McDonald M.L., Juan W.T., Huang C.J., Lai Y.T., Lo W.S., Chen C.F. (2012). The chicken frizzle feather is due to an α-keratin (KRT75) mutation that causes a defective rachis. PLoS Genet..

[23] Wright D., Boije H., Meadows J.R., Bed’hom B., Gourichon D., Vieaud A., Tixier-Boichard M., Rubin C.J., Imsland F., Hallbook F. (2009). Copy number variation in intron 1 of SOX5 causes the Pea-comb phenotype in chickens. PLoS Genet..

[24] Dorshorst B., Harun-Or-Rashid M., Bagherpoor A.J., Rubin C.J., Ashwell C., Gourichon D., Tixier-Boichard M., Hallbook F., Andersson L. (2015). A genomic duplication is associated with ectopic eomesodermin expression in the embryonic chicken comb and two duplex-comb phenotypes. PLoS Genet..

[25] Imsland F., Feng C., Boije H., Bed’hom B., Fillon V., Dorshorst B., Rubin C.J., Liu R., Gao Y., Gu X. (2012). The Rose-comb mutation in chickens constitutes a structural rearrangement causing both altered comb morphology and defective sperm motility. PLoS Genet..

[26] Li D., Li Y., Li M., Che T., Tian S., Chen B., Zhou X., Zhang G., Gaur U., Luo M. (2019). Population genomics identifies patterns of genetic diversity and selection in chicken. BMC Genom..

[27] Wang H., Wen J., Li H., Zhu T., Zhao X., Zhang J., Zhang X., Tang C., Qu L., Gemingguli M. (2022). Candidate pigmentation genes related to feather color variation in an indigenous chicken breed revealed by whole genome data. Front. Genet..

[28] Huang X., Otecko N.O., Peng M., Weng Z., Li W., Chen J., Zhong M., Zhong F., Jin S., Geng Z. (2020). Genome-wide genetic structure and selection signatures for color in 10 traditional Chinese yellow-feathered chicken breeds. BMC Genom..

[29] Guo Y., Gu X., Sheng Z., Wang Y., Luo C., Liu R., Qu H., Shu D., Wen J., Crooijmans R.P. (2016). A Complex Structural Variation on Chromosome 27 Leads to the Ectopic Expression of HOXB8 and the Muffs and Beard Phenotype in Chickens. PLoS Genet..

[30] Yang S., Shi Z., Ou X., Liu G. (2019). Whole-genome resequencing reveals genetic indels of feathered-leg traits in domestic chickens. J. Genet..

[31] Bortoluzzi C., Megens H.J., Bosse M., Derks M.F.L., Dibbits B., Laport K., Weigend S., Groenen M.A.M., Crooijmans R. (2020). Parallel Genetic Origin of Foot Feathering in Birds. Mol. Biol. Evol..

[32] Jiang H., Yang D., Ma Z., Xun W., Hou G., Shi L. (2022). Genome-wide Association Study of Meat Color Traits in Danzhou Chickens. Chin. J. Anim. Sci..

[33] Tan X., Liu R., Zhao D., He Z., Li W., Zheng M., Li Q., Wang Q., Liu D., Feng F. Large-scale genomic and transcriptomic analyses elucidate the genetic basis of high meat yield in chickens. J. Adv. Res..

[34] Cai D., Wang Z., Zhou Z., Lin D., Ju X., Nie Q. (2023). Integration of transcriptome sequencing and whole genome resequencing reveal candidate genes in egg production of upright and pendulous-comb chickens. Poult. Sci..

[35] Dong X., Li J., Zhang Y., Han D., Hua G., Wang J., Deng X., Wu C. (2019). Genomic Analysis Reveals Pleiotropic Alleles at EDN3 and BMP7 Involved in Chicken Comb Color and Egg Production. Front. Genet..

[36] Zhang Q., Zhu F., Liu L., Zheng C.W., Wang D.H., Hou Z.C., Ning Z.H. (2015). Integrating transcriptome and genome re-sequencing data to identify key genes and mutations affecting chicken eggshell qualities. PLoS ONE.

[37] Ren T., Li W., Liu D., Liang K., Wang X., Li H., Jiang R., Tian Y., Kang X., Li Z. (2019). Two insertion/deletion variants in the promoter region of the QPCTL gene are significantly associated with body weight and carcass traits in chickens. Anim. Genet..

[38] Liu D., Han R., Wang X., Li W., Tang S., Li W., Wang Y., Jiang R., Yan F., Wang C. (2019). A novel 86-bp indel of the motilin receptor gene is significantly associated with growth and carcass traits in Gushi-Anka F(2) reciprocal cross chickens. Br. Poult. Sci..

[39] An B., Xia J., Chang T., Wang X., Xu L., Zhang L., Gao X., Chen Y., Li J., Gao H. (2019). Genome-wide association study reveals candidate genes associated with body measurement traits in Chinese Wagyu beef cattle. Anim. Genet..

[40] Wu Z., Derks M.F.L., Dibbits B., Megens H.J., Groenen M.A.M., Crooijmans R. (2018). A Novel Loss-of-Function Variant in Transmembrane Protein 263 (TMEM263) of Autosomal Dwarfism in Chicken. Front. Genet..

[41] Shi S., Shao D., Yang L., Liang Q., Han W., Xue Q., Qu L., Leng L., Li Y., Zhao X. (2023). Whole genome analyses reveal novel genes associated with chicken adaptation to tropical and frigid environments. J. Adv. Res..

[42] Chen B. (2018). Genomic Resequencing Reveals Genetic Diversity and Evolutionary Selection Patterns in Chickens. Ph.D. Thesis.

[43] Khatri B., Hayden A.M., Anthony N.B., Kong B.C. (2018). Whole Genome Resequencing of Arkansas Progressor and Regressor Line Chickens to Identify SNPs Associated with Tumor Regression. Genes.

[44] Popovic D., Vucic D., Dikic I. (2014). Ubiquitination in disease pathogenesis and treatment. Nat. Med..

[45] Dey S., Parveen A., Tarrant K.J., Licknack T., Kong B.C., Anthony N.B., Rhoads D.D. (2018). Whole genome resequencing identifies the CPQ gene as a determinant of ascites syndrome in broilers. PLoS ONE.

[46] Guo X., Li Y.Q., Wang M.S., Wang Z.B., Zhang Q., Shao Y., Jiang R.S., Wang S., Ma C.D., Murphy R.W. (2018). A parallel mechanism underlying frizzle in domestic chickens. J. Mol. Cell Biol..

[47] Noorai R.E., Shankar V., Freese N.H., Gregorski C.M., Chapman S.C. (2019). Discovery of genomic variations by whole-genome resequencing of the North American Araucana chicken. PLoS ONE.

[48] Liu X., Liu L., Wang J., Cui H., Chu H., Bi H., Zhao G., Wen J. (2020). Genome-Wide Association Study of Muscle Glycogen in Jingxing Yellow Chicken. Genes.

[49] Kong H.R., Anthony N.B., Rowland K.C., Khatri B., Kong B.C. (2018). Genome re-sequencing to identify single nucleotide polymorphism markers for muscle color traits in broiler chickens. Asian-Australas. J. Anim. Sci..

[50] Boschiero C., Moreira G.C.M., Gheyas A.A., Godoy T.F., Gasparin G., Mariani P., Paduan M., Cesar A.S.M., Ledur M.C., Coutinho L.L. (2018). Genome-wide characterization of genetic variants and putative regions under selection in meat and egg-type chicken lines. BMC Genom..

[51] Wang Q., Li D., Guo A., Li M., Li L., Zhou J., Mishra S.K., Li G., Duan Y., Li Q. (2020). Whole-genome resequencing of Dulong Chicken reveal signatures of selection. Br. Poult. Sci..

[52] Wang M.S., Thakur M., Peng M.S., Jiang Y., Frantz L.A.F., Li M., Zhang J.J., Wang S., Peters J., Otecko N.O. (2020). 863 genomes reveal the origin and domestication of chicken. Cell Res..

[53] Wang M.S., Li Y., Peng M.S., Zhong L., Wang Z.J., Li Q.Y., Tu X.L., Dong Y., Zhu C.L., Wang L. (2015). Genomic Analyses Reveal Potential Independent Adaptation to High Altitude in Tibetan Chickens. Mol. Biol. Evol..

[54] Weng Z., Xu Y., Zhong M., Li W., Chen J., Zhong F., Du B., Zhang B., Huang X. (2022). Runs of homozygosity analysis reveals population characteristics of yellow-feathered chickens using re-sequencing data. Br. Poult. Sci..

[55] Ren P., Deng F., Chen S., Ran J., Li J., Yin L., Wang Y., Yin H., Zhu Q., Liu Y. (2021). Whole-genome resequencing reveals loci with allelic transmission ratio distortion in F(1) chicken population. Mol. Genet. Genom..

